# Supplementary material for: iSeqSearch: incremental protein search for iBlast/iMMSeqs2/iDiamond
Source: PeerJ. 2025 Apr 28;13:e19171. doi: 10.7717/peerj.19171 (PMC12045279; doi:10.7717/peerj.19171)
Supplement: Supplemental Information 1 [file peerj-13-19171-s001.pdf]

Supplementary Materials

1 Experimental Settings

1. Experiment Objective: To discern the differences between using an incremental method and not, by verifying that similar results can be achieved with the incremental approach.
2. Dataset Selection: The SCOPe Astral dataset[1] will be employed, comprising 305,543 sequences categorized into 7 SCOPe protein classes such as class a,b,c,d,e,f, and g. (astral-scopedom-seqres-gd-all-2.08-2023-01-06: latest version)

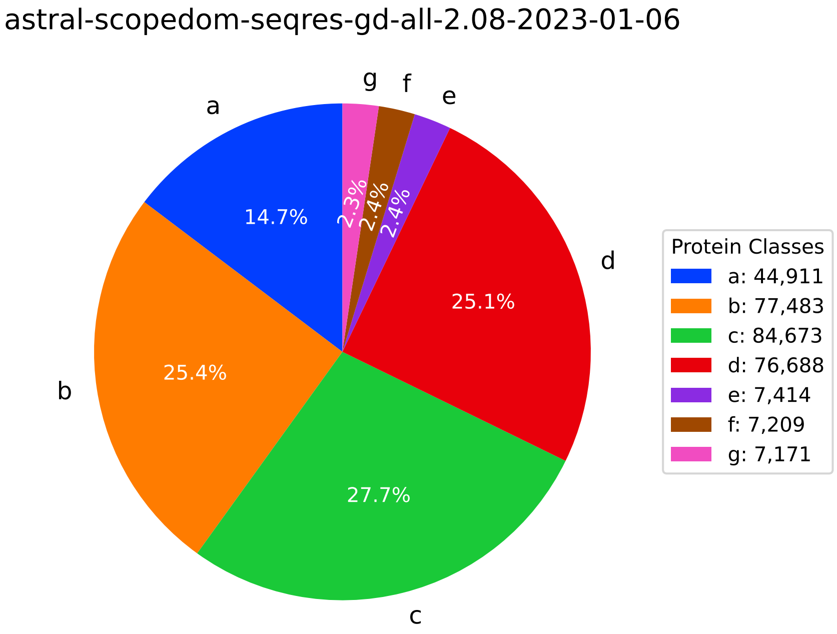

Figure 1. Protein Class Distribution

3. Experimental Design: The dataset will be divided into 10 batches. 1st batch is stratified random sampled and other 9 batches are random sampled based on protein class. A stratified randomly chosen 1st batch will serve as the query, while the remaining 9 batches will be sequentially combined for the search experiments. The experiments will be conducted using the default settings for BLASTP, MMseqs2, and DIAMOND. Only the number of threads was set to 32; all other settings were default.

2 SCOPe dataset

This project utilizes the SCOPe Astral dataset, which includes the extended database for the structural classification of proteins (SCOPe, Structural Classification of Proteins — extended). The features of this dataset are as follows: It consists of a total of 305,543 sequences from 4,327 species, categorized into 7 SCOPe protein classes, 1,257 protein folds, 2,065 protein superfamilies, and 5,084 protein families. The data, accessible via the SCOPe website, notably through the file 'astral-scopedom-seqres-gd-all-2.08-2023-01-06.fa', offers comprehensive information on protein structures and classifications, representing a rich resource for researchers exploring the vast diversity and complexity of biological systems.

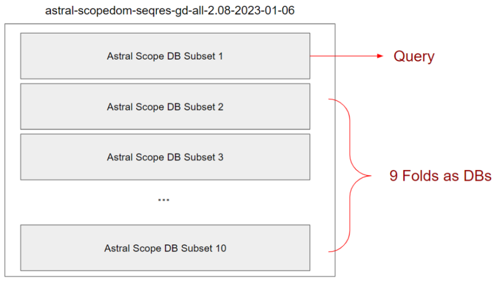

(a) Step 1: Data set is divided by 10 batches. Query has 30558 sequences.

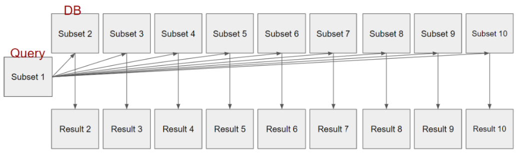

(b) Step 2: Get partial results for the results of incremental methods.

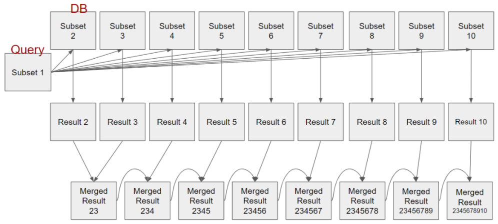

(c) Step 3: Get results from incremental methods.

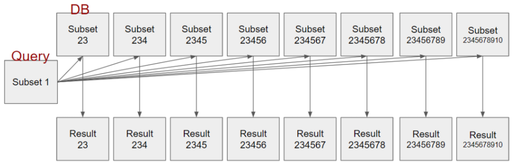

(d) Step 4: Get results from non-incremental methods.

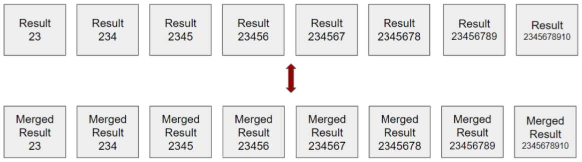

(e) Step 5: Comparing incremental methods and non-incremental methods.

Figure 2. Illustration of the process for experiments

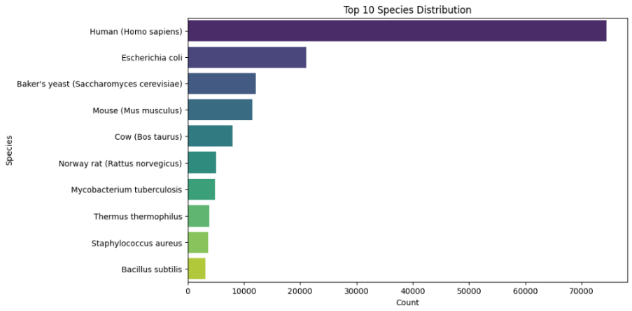

Figure 3. Species Distribution

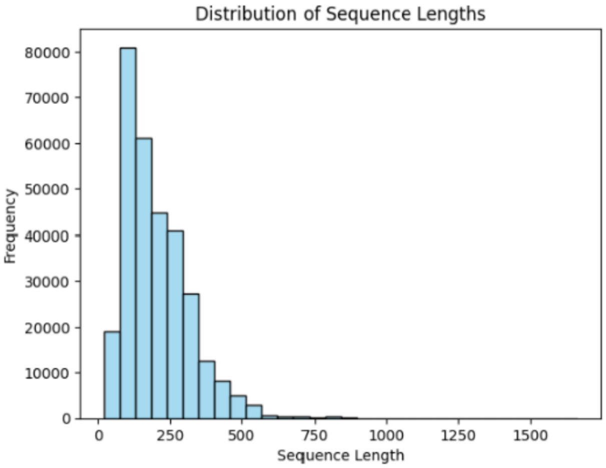

Figure 4. Sequence Length Distribution

3 Time Measure

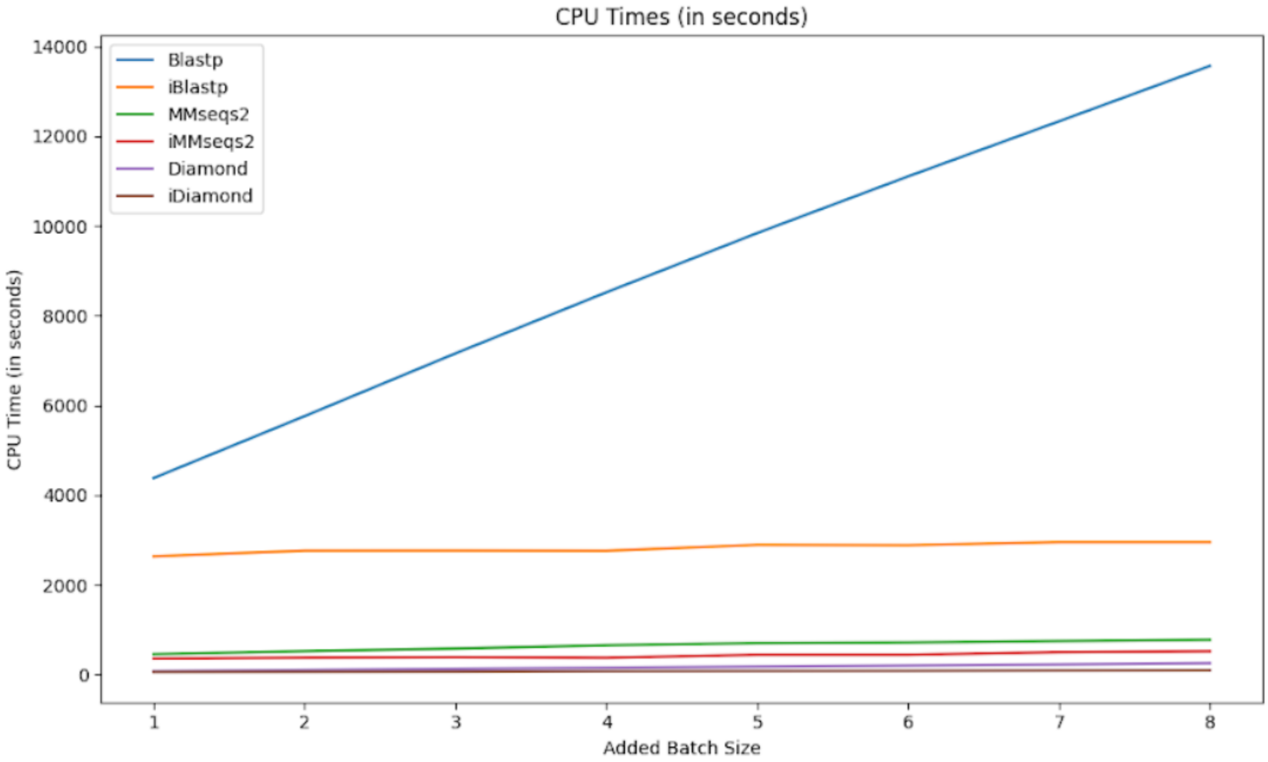

Figure 5. Time Comparision

The times for incremental methods represent the search time for the newly added batch and the time to merge these results with the previous results. For non-incremental methods, the times represent the search time for the entire database. The database consists of nine out of the ten portions of the entire Astral Scope dataset. In the experiments, one of the ten portions is used as the query, and the remaining batches from 2 to 10 are incrementally combined to test the search results. The time differences between MMSeqs2 and iMMSeqs2, as well as between Diamond and iDiamond, appear relatively small compared to those observed between BLAST and iBlast, as shown in Figure 5 of the supplementary material. This is because MMSeqs2 and Diamond are already significantly faster than BLAST, making the time savings provided by the incremental method less pronounced for these tools. Nevertheless, Figure 1 in the main paper clearly demonstrates that the incremental methods, iMMSeqs2 and iDiamond, still offer noticeable speed improvements, underscoring their efficiency.

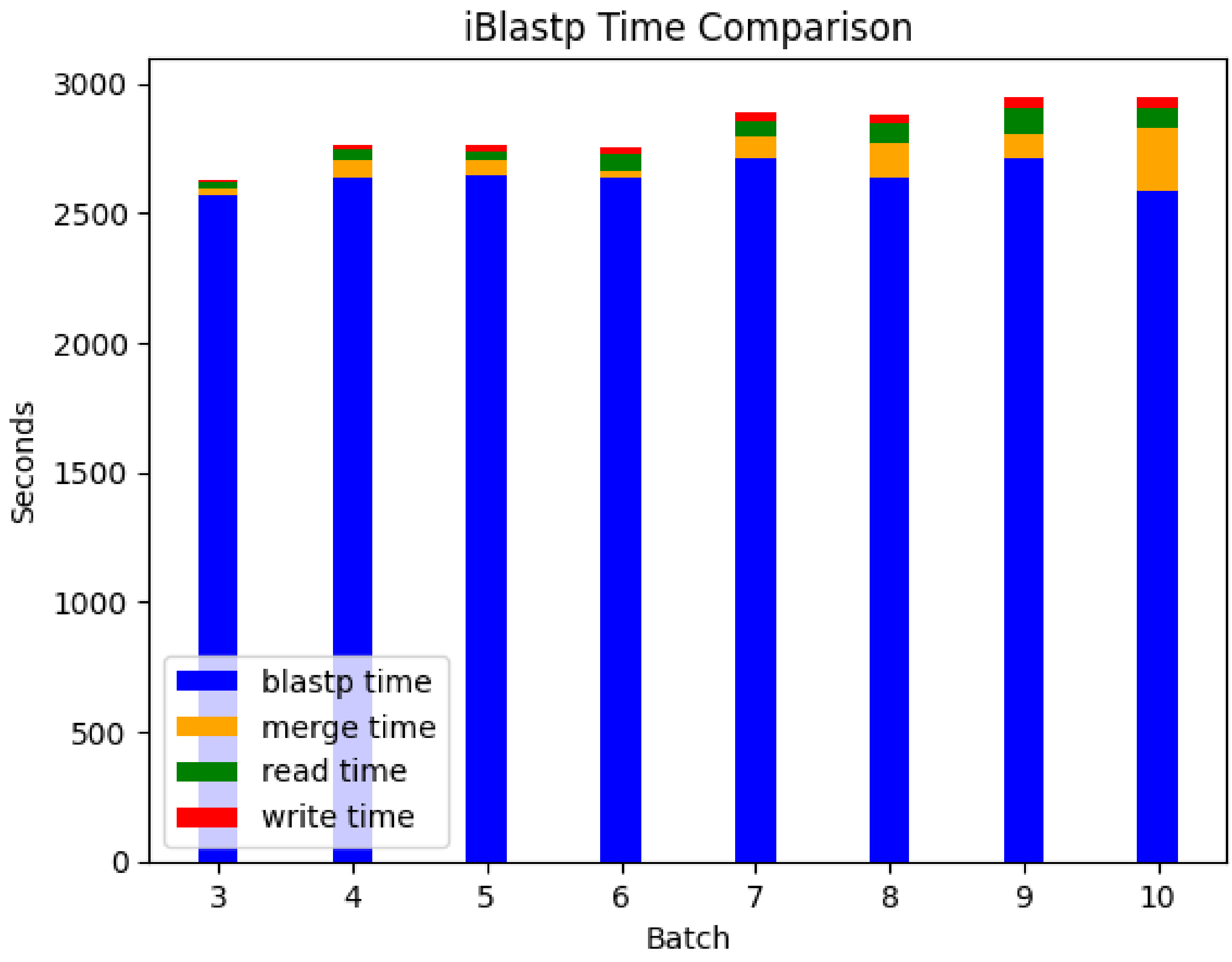

Figure 6. iBlast Time Comparison

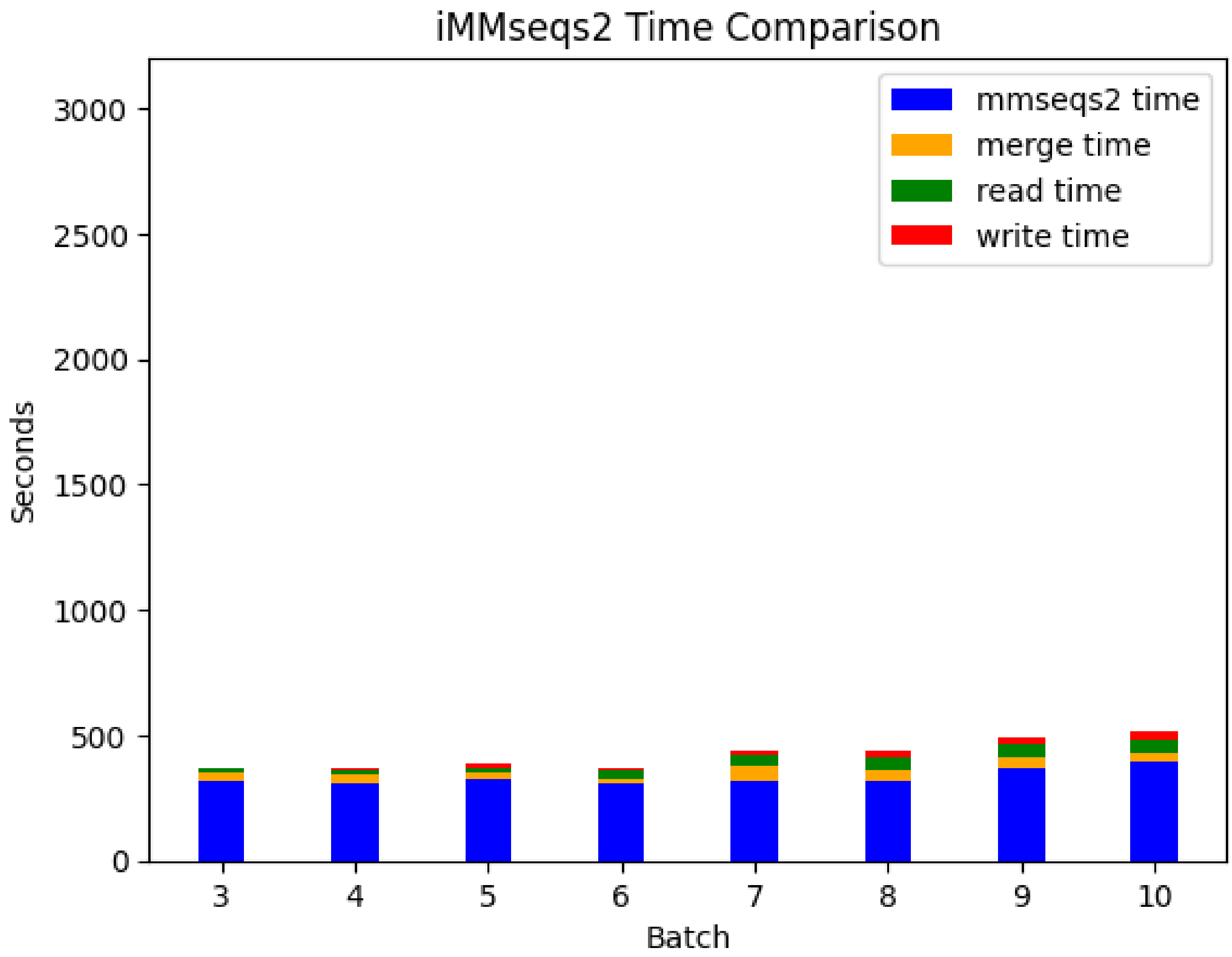

Figure 7. iMMseqs2 Time Comparison

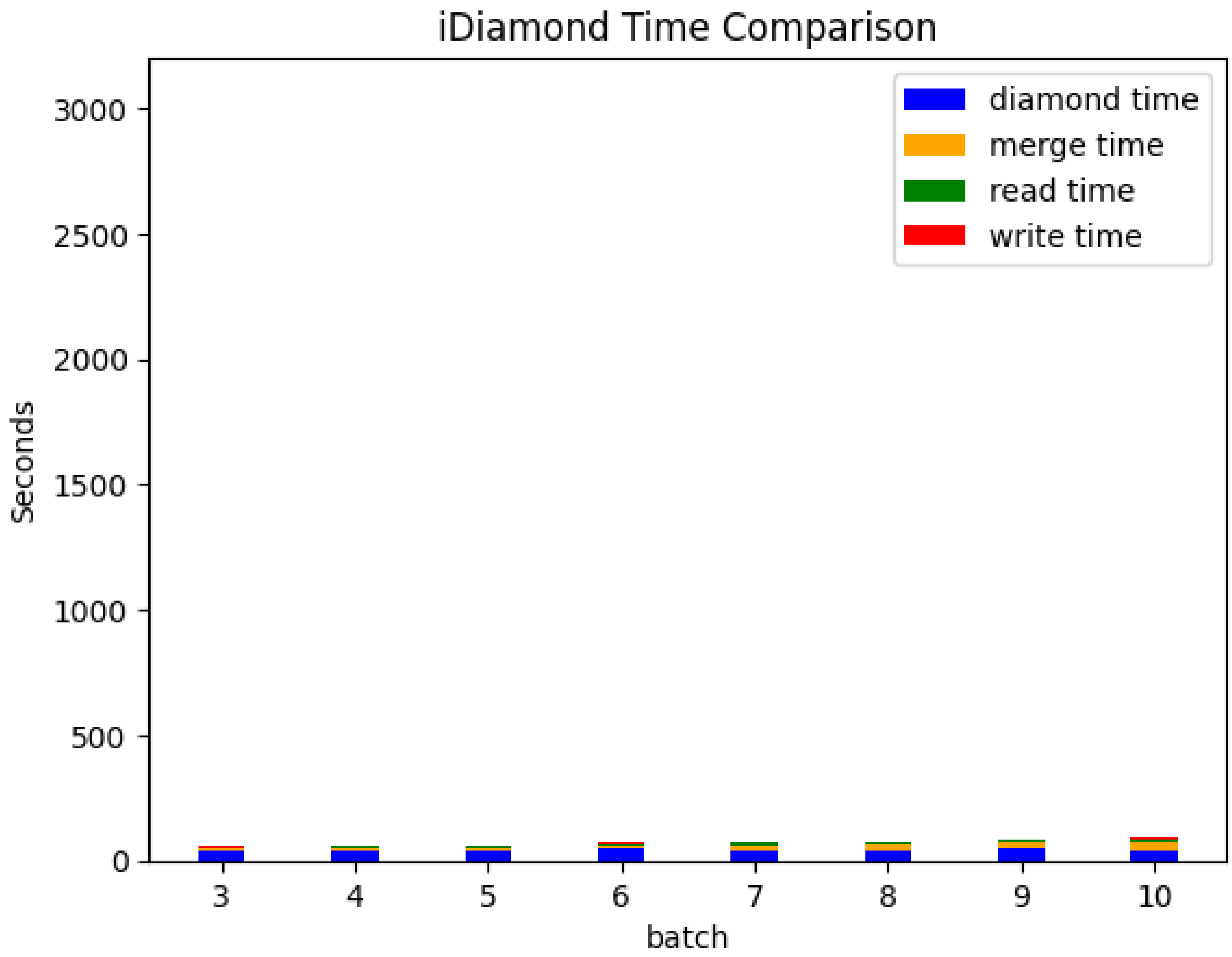

Figure 8. iDiamond Time Comparison

4 Protein SCOPe-Class Classification

We aimed to see how well we could classify the queries into their 7 classes (see Fig. 1) after the training of each random sub-batches. The graphs below compare the protein class f1 score of non-incremental methods and incremental methods based on the e-value top hit criterion. The results of the incremental experiments show a trend of increasing f1 score across all cases. The classification linearly increases as data is added in all cases up to 99% when all SCOPe[1] classes are known. In the last batch, every training class was included, leading to an increase in performance and achieving good results. The ratio of queries to the last batches is similar.

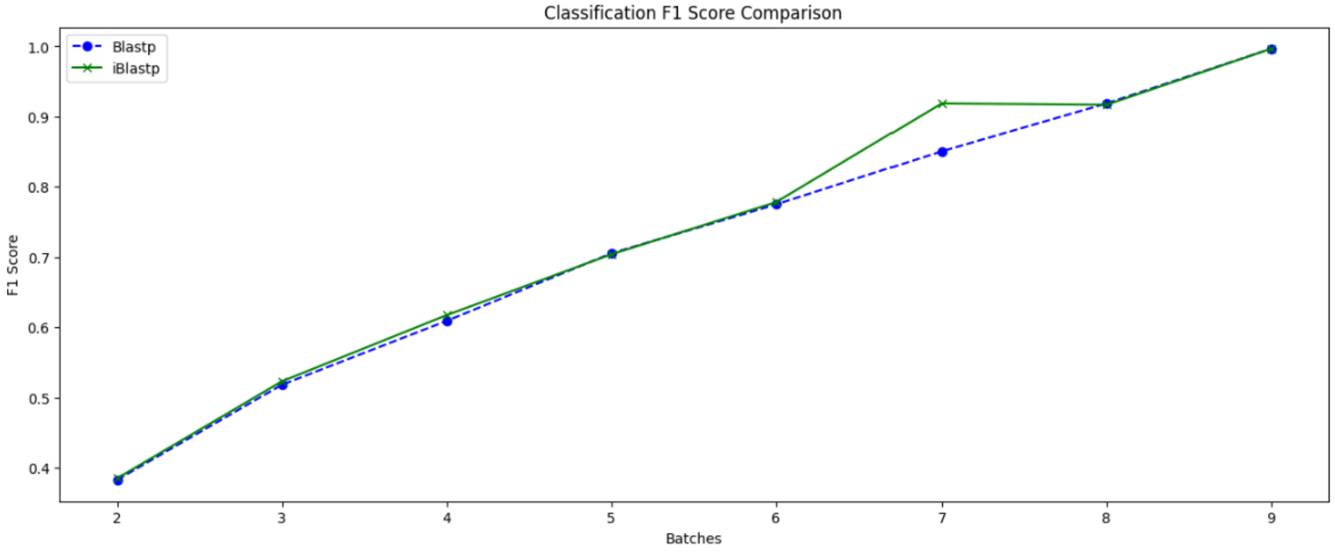

Figure 9. Blastp vs iBlastp

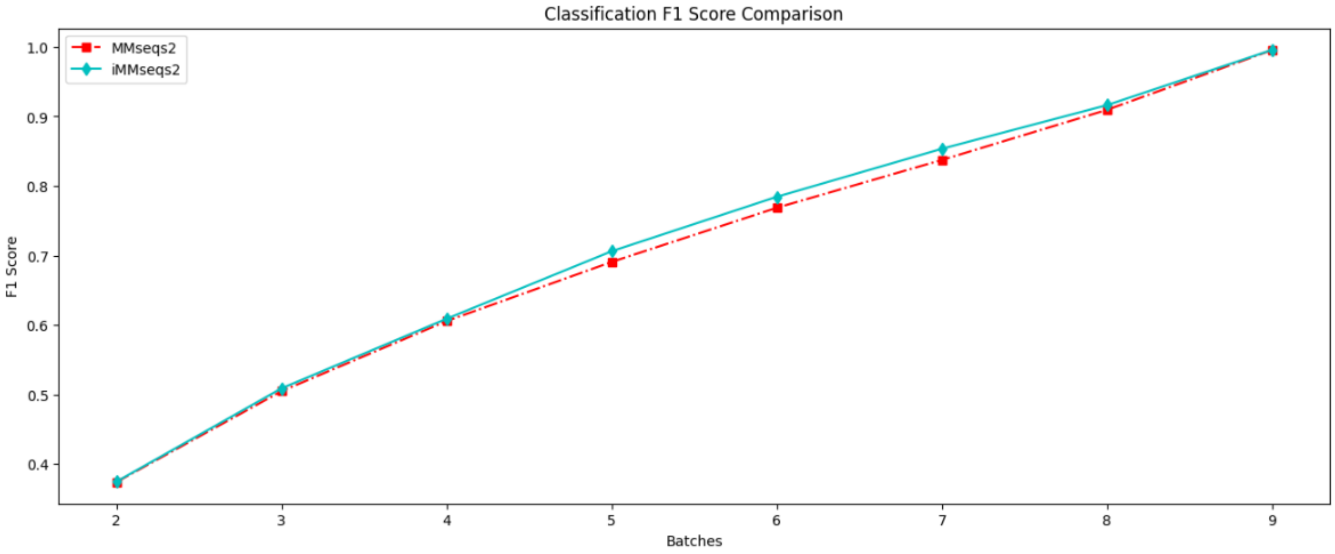

Figure 10. MMseqs2 vs iMMseqs2

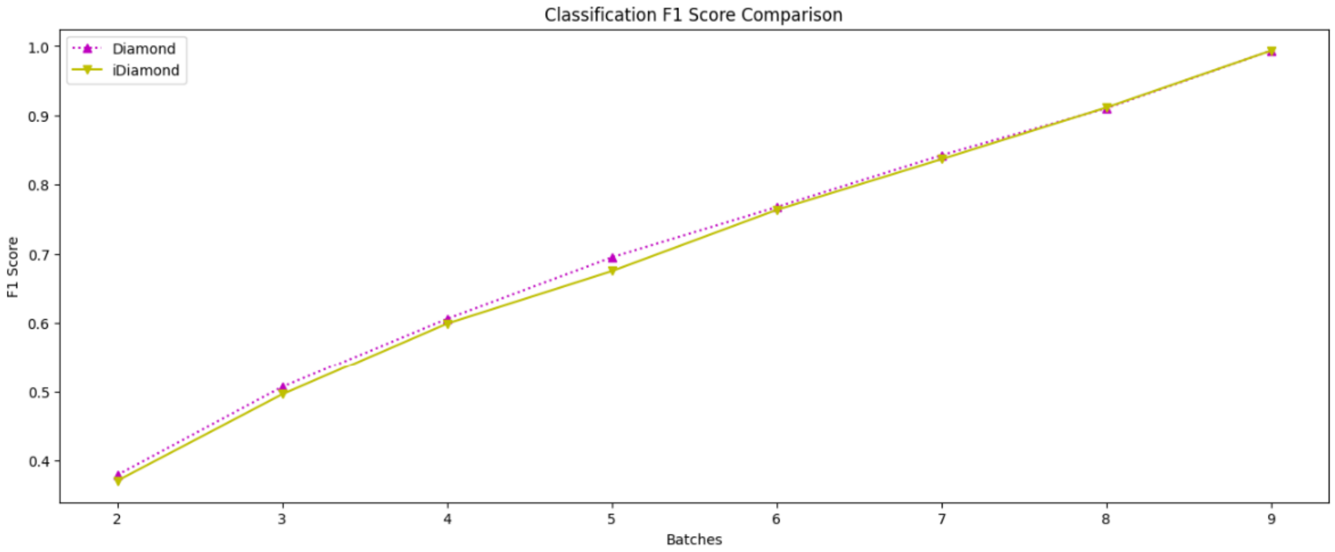

Figure 11. Diamond vs iDiamond

5 Venn Diagram with the hit number ratio

The Venn diagrams from the second and final batches illustrates that most of the hits from the non-incremental methods are included in the incremental methods. Specifically, the hits from Blastp are mostly included in the hits from iBlastp, and the hits from Diamond are also included in the hits from iDiamond. Similarly, the hits from MMseqs2 are included in the hits from iMMseqs2.

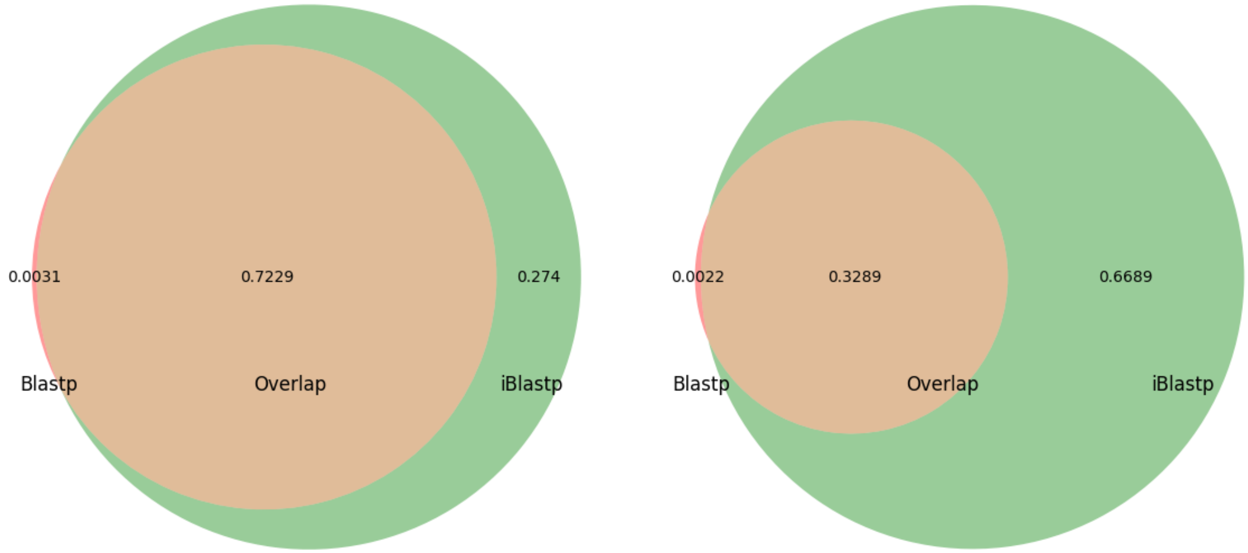

Figure 12. Blastp vs iBlastp with 2nd batch and last batch

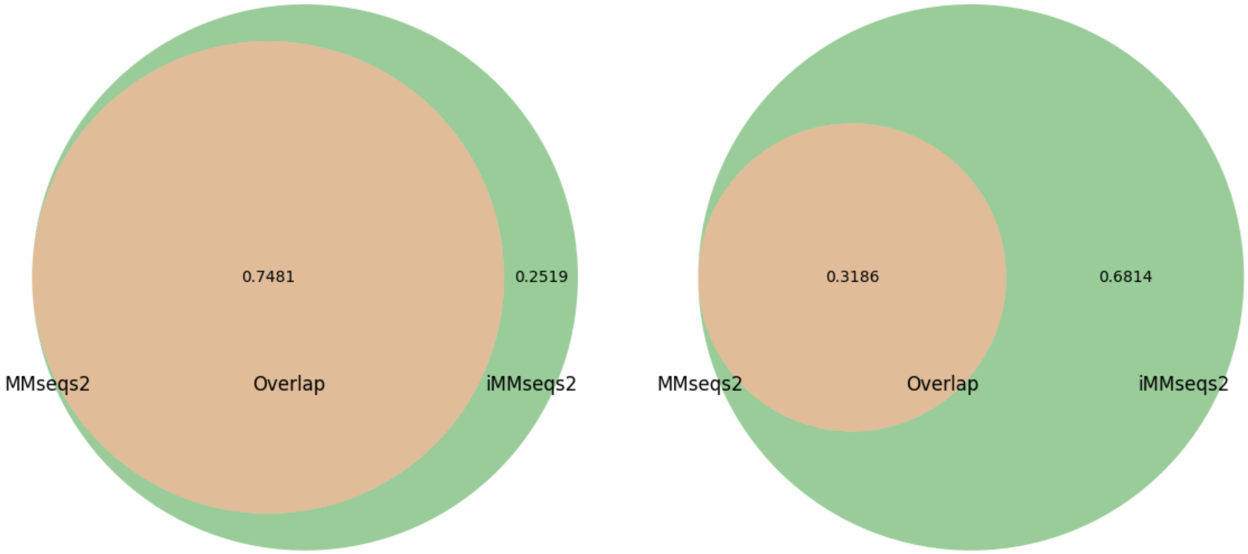

Figure 13. MMseqs2 vs iMMseqs2 with 2nd batch and last batch

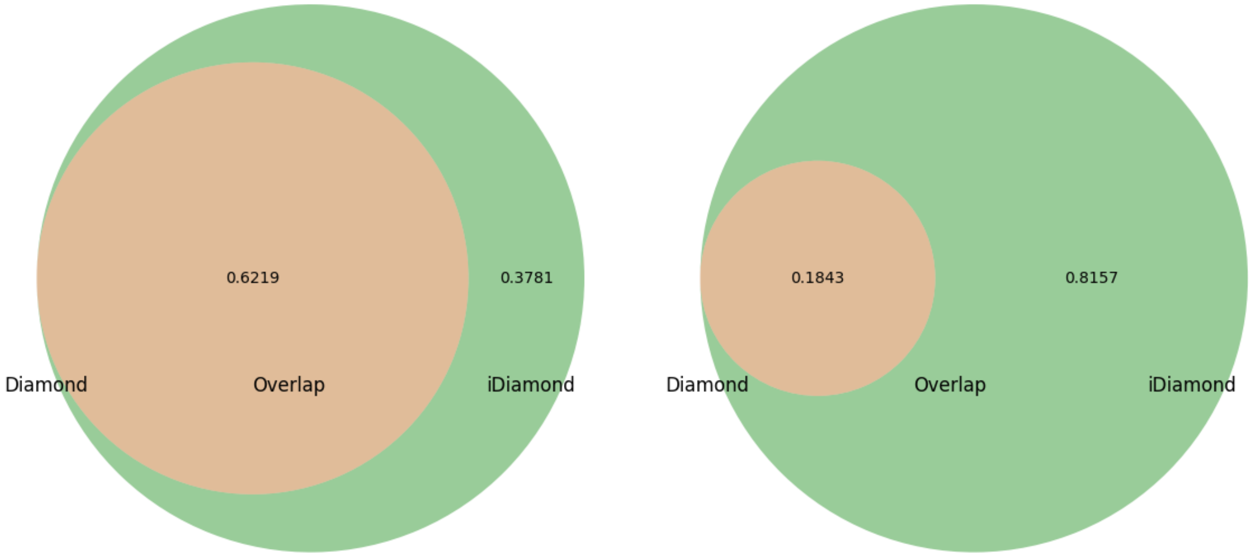

Figure 14. Diamond vs iDiamond with 2nd batch and last batch

6 No-hit per query ratio (non-stratified)

The figure below shows the proportion of queries that do not have a single hit as the search progresses incrementally. It can be seen that the incremental methods have a lower proportion of queries with no hits compared to the non-incremental methods. This indicates that incremental methods not only provide more hits but also cover a larger number of queries with hits than non-incremental methods. The first row at the top indicates the size of the increased batch.

	2	3	4	5	6	7	8	9
Blastp	0.0019	0.0018	0.0018	0.0013	0.0012	0.0012	0.0010	0.0010
iBlastp	0.0006	0.0001	0.0000	0.0000	0.0000	0.0000	0.0000	0.0000
MMseqs2	0.0248	0.0170	0.0127	0.0095	0.0079	0.0067	0.0056	0.0049
iMMseqs2	0.0247	0.0169	0.0124	0.0092	0.0076	0.0063	0.0053	0.0047
Diamond	0.0387	0.0266	0.0199	0.0154	0.0126	0.0106	0.0088	0.0075
iDiamond	0.0387	0.0265	0.0198	0.0154	0.0126	0.0106	0.0088	0.0075

Figure 15. This graph shows the ratio of no hit in query when batch increase

7 DCG measure

This table compares the DCG of incremental and non-incremental methods. The incremental method demonstrates superior DCG across all batches. The first row at the top indicates the size of the increased batch.

	2	3	4	5	6	7	8	9
Blastp	1275.4489	1611.0705	1886.7882	2117.4993	2327.6501	2513.8179	2677.9734	2829.0995
iBlastp	1381.0740	1854.8394	2296.3123	2716.6527	3122.8251	3516.9319	3901.4425	4277.6957
MMseqs2	1304.7009	1618.7234	1876.5754	2088.6984	2270.1146	2417.2935	2544.9096	2658.6221
iMMseqs2	1413.4290	1880.2749	2314.3664	2725.1164	3121.0765	3504.8746	3879.8322	4243.5533
Diamond	709.1630	774.2682	816.7293	847.9597	872.7081	891.9916	908.1241	922.0935
iDiamond	907.8142	1179.0332	1426.2112	1658.1434	1879.5196	2092.3212	2299.8423	2500.8473

Figure 16. Log DCG comparison for each batches

Log Discounted Cumulative Gain (log DCG) is a standard metric for quantitatively evaluating the quality of search results, focusing on ranking performance. However, it is not intended to measure biological relevance.

8 Case study(Protein family) : non-incremental method vs incremental method

The blast analysis has revealed duplicated hits. To ensure a fair comparison, particularly in the context of Blastp analysis, it is crucial to address these duplications and apply a stringent filter for E-values. In Blastp methodology, it has not removed these duplications nor filtered out hits with higher E-values. Therefore, to enhance the accuracy and reliability of the comparative analysis, we removed duplicated entries, retained only the hits with the smallest E-values, and discarded those with E-values greater than 1e-5, without limiting the number of hits. This adjustment will help maintain the integrity of our findings and ensure that the results are both robust and meaningful.

Query case 'd2ap2c1(right label: b.1.1.1)' and 'd6iyia_(a.1.1.0)' are used for this case study. One protein was randomly selected from the SCOPe alpha protein class and another from the beta protein class. These proteins were chosen to ensure diverse structural representation and to assess the algorithm's performance across different protein folds.

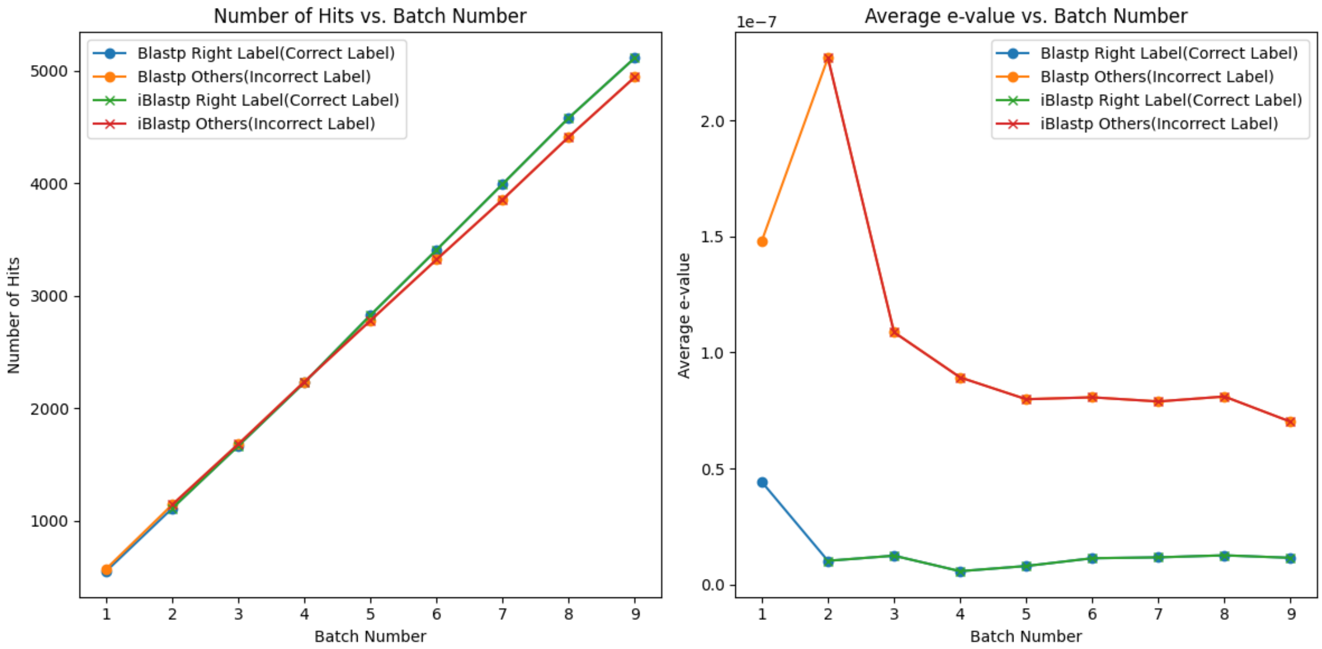

Figure 17. Query "d2ap2c1" Case Study: Blastp vs iBlastp where “Right label" is the Correct Protein Family label and others mean all incorrect labels.

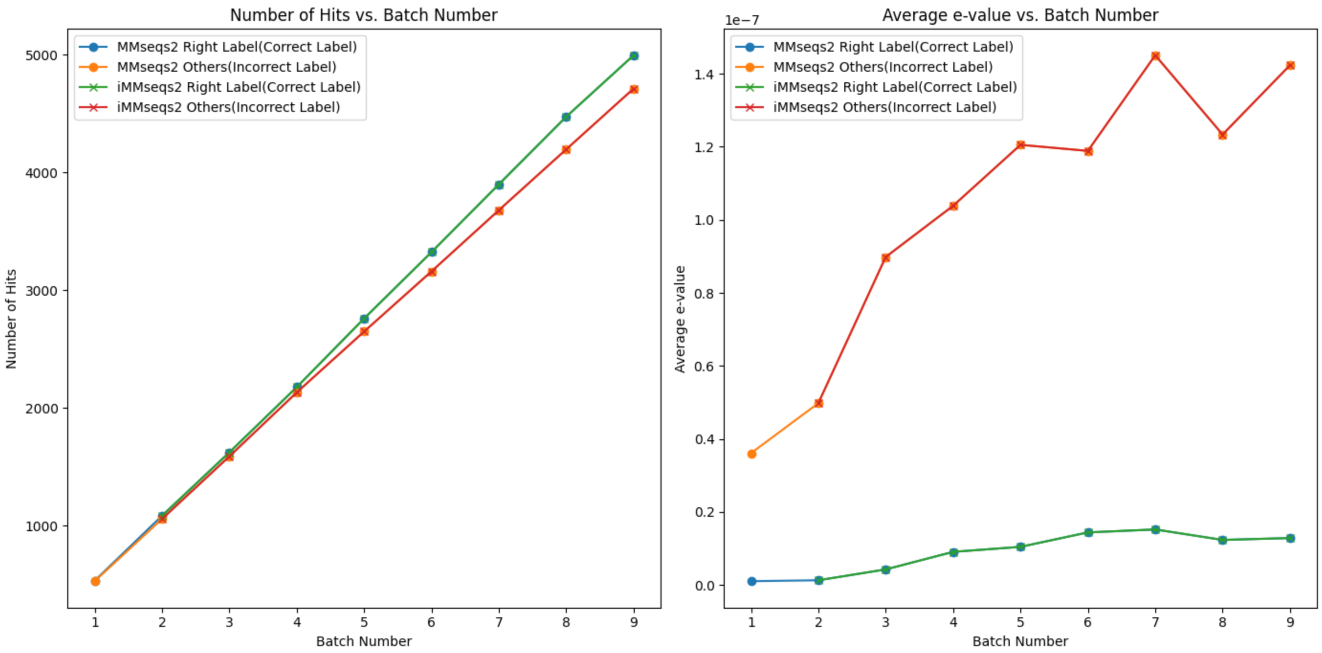

Figure 18. Query "d2ap2c1" Case Study: MMseqs2 vs iMMseqs2 where “Right label" is the Correct Protein Family label and others mean all incorrect labels.

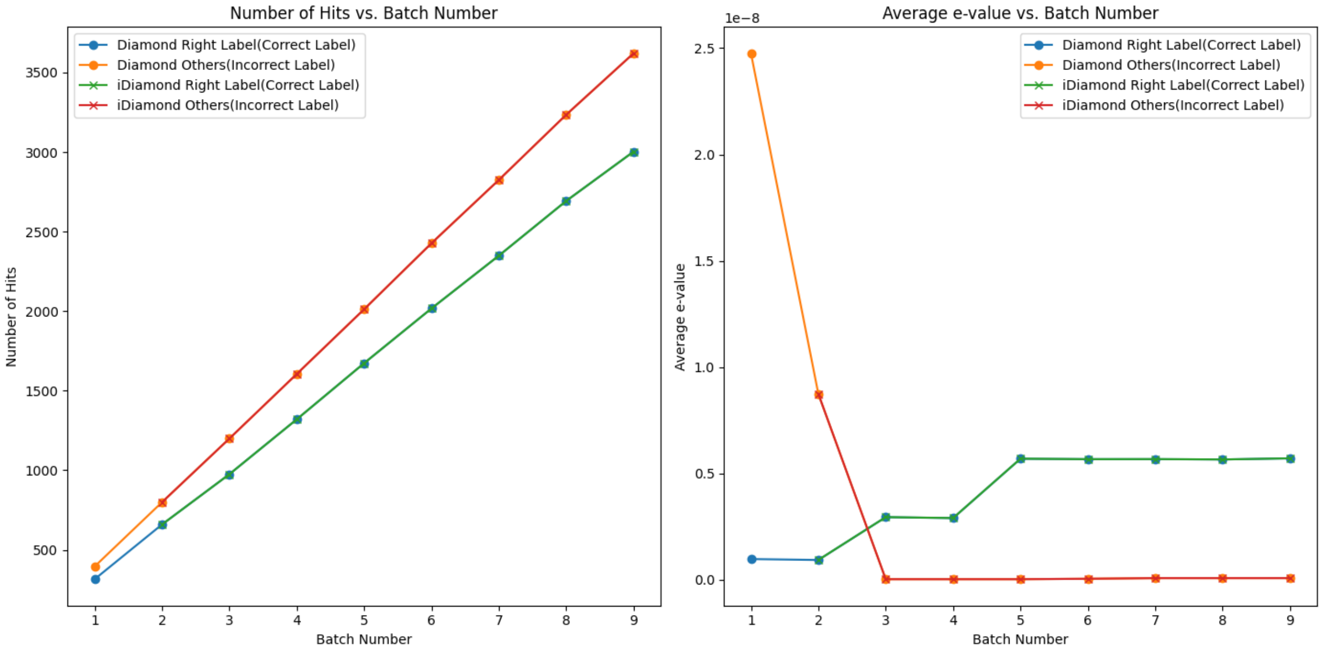

Figure 19. Query "d2ap2c1" Case Study: Diamond vs iDiamond where “Right label" is the Correct Protein Family label and others mean all incorrect labels.

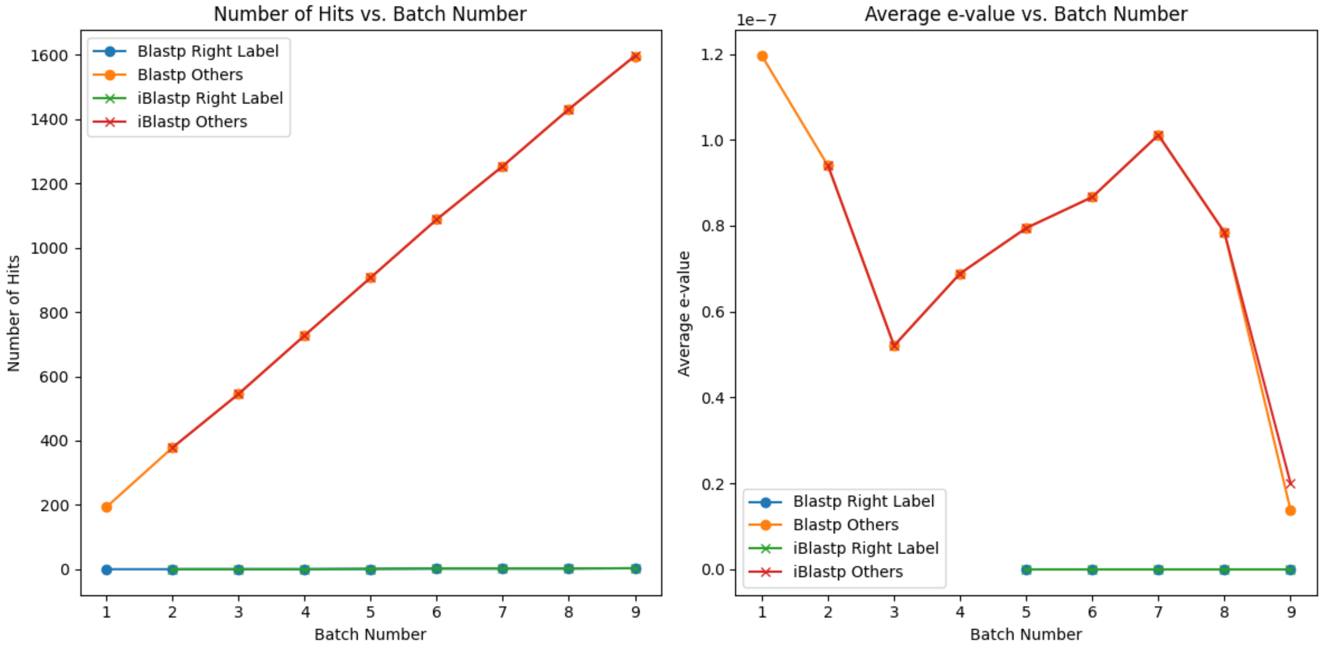

Figure 20. Query "d6iyia_" Case Study: Blastp vs iBlastp where “Right label" is the Correct Protein Family label and others mean all incorrect labels.

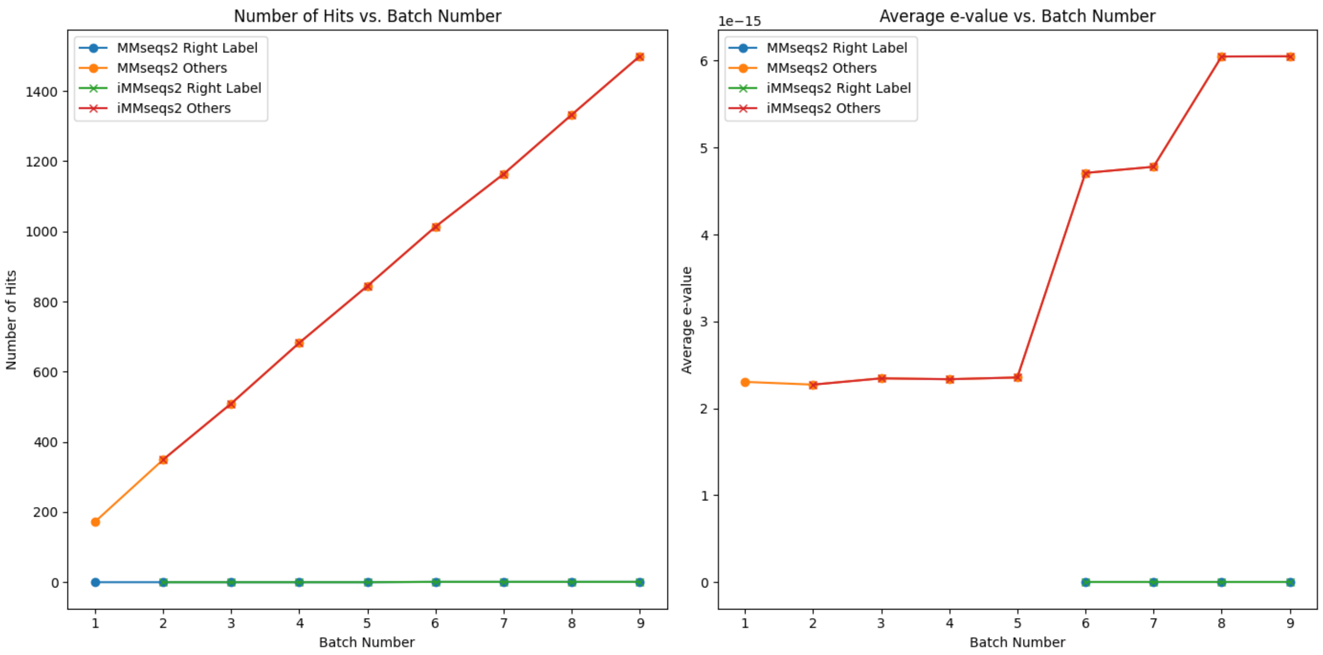

Figure 21. Query "d6iyia_" Case Study: MMseqs2 vs iMMseqs2 where “Right label" is the Correct Protein Family label and others mean all incorrect labels.

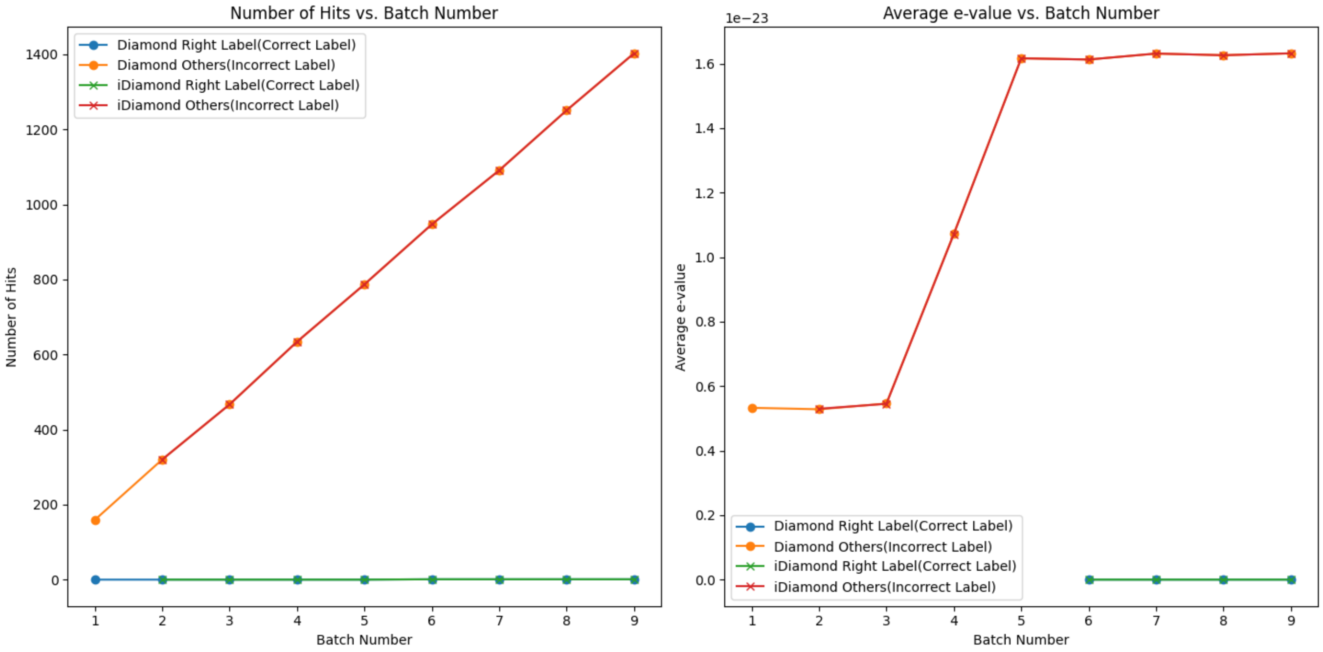

Figure 22. Query "d6iyia_" Case Study: Diamond vs iDiamond where “Right label" is the Correct Protein Family label and others mean all incorrect labels.

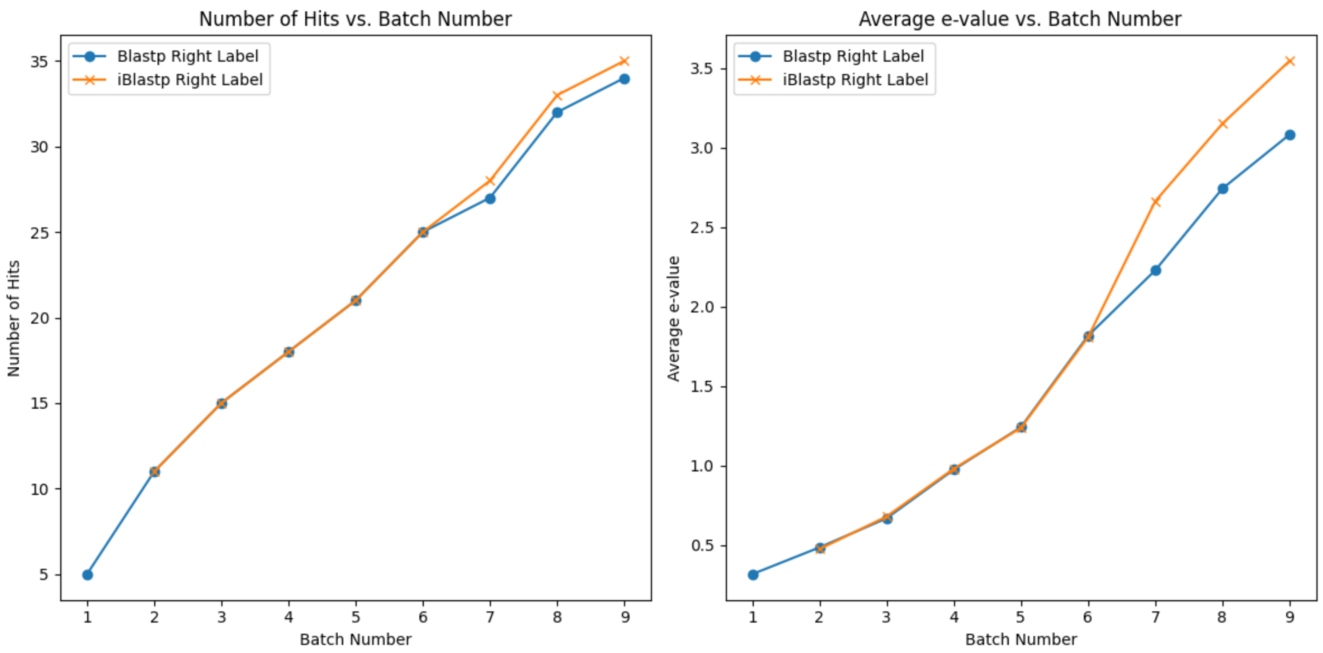

Figure 23. Query "d6iyia_" Case Study: Blastp vs iBlastp without 1e-5 threshold where “Right label" is the Correct Protein Family label.

This experiment is conducted with specific conditions. The hit number limit was removed, and duplicates were handled by retaining the hit with the smallest e-value, excluding results with an e-value greater than or equal to 1e-5. The resulting graph above reflects these conditions. The number of hits between incremental methods (iBlastp, iMMseqs2 and iDiamond) and non-incremental methods (Blastp, MMseqs2, Diamond) remained similar even as the batch size increased. The average e-value also showed very similar results. Additionally, without 1e-5 threshold, iBlastp shows more hits and higher average e-value compared to Blastp like Figure 23.

This case study highlights the potential biological significance of using incremental methods. For instance, the chosen query proteins, d2ap2c1 and d6iyia_, represent distinct structural classes (beta and alpha, respectively), providing an opportunity to evaluate the algorithm's ability to detect biologically meaningful hits across diverse protein folds. The findings suggest that incremental methods, while generating a similar number of hits compared to non-incremental methods, offer flexibility in adapting to larger datasets and varying batch sizes. Furthermore, the higher number of hits detected without the 1e-5 threshold indicates the possibility of identifying sequences that, although statistically less significant, may still have biological relevance in certain contexts, such as rare protein families or environmental datasets.

9 Additional Experiments: Swiss Prot Database with Southern California wastewater

The dataset utilized for this experiment consists of 723,864 spots(single sequence among paired sequences) from sample SRR19607378. This dataset originates from the longitudinal metatranscriptomic sequencing of Southern California wastewater. The data, under accession numbers PRJNA729801, provides for studying wastewater microbial communities and their characteristics.

The Swiss Prot database was selected for its comprehensive collection of manually annotated and reviewed protein sequences. The following versions were used: Release 2.0 (the first version from 2005), 2010_01, 2015_01, 2020_01, and the latest release, 2024_05.

To ensure the integrity and consistency of the experiments across the SwissProt database versions, a meticulous process was followed due to the inherent complexity of the data. The challenges arose primarily because some sequences were either deleted or changed between versions. To address this, the approach involved processing the data in reverse chronological order, starting from the latest version and working backward.

By removing deleted sequences from the previous version first, potential conflicts or redundancies could be avoided. Similarly, for sequences marked as changed, they were updated in the previous version to reflect the modifications accurately. This reverse-order method was crucial to ensure that no discrepancies or oversights occurred during the alignment of the datasets. While this approach added complexity to the process, it was necessary to maintain consistency and avoid the risk of inadvertently overlooking changes or deletions.

The relative abundance of the top 10 dominant, as determined using MMseqs2 and iMMseqs2, is visualized in Figure 24 and Figure 25, respectively. These figures provide a detailed comparison of species proportions across the databases from 2005, 2010, 2015, 2020, and 2024. Each database represents a unique snapshot in time, reflecting the evolving coverage and curation of annotated protein sequences within SwissProt.

Figures 24 and 25 compare the relative abundance of the top 10 dominant species identified using MMseqs2 and iMMseqs2, respectively, across SwissProt database versions from 2005 to 2024. Both analyses exhibit highly consistent trends, with significant overlap in the identified dominant species and their proportions. For example, Tobacco rattle virus (strain SYM) consistently ranks as the most abundant species in 2005 in both methods, followed by Escherichia coli (strain K12) and Pseudomonas aeruginosa (strain ATCC 15692). Similarly, in the later database versions (2020 and 2024), Tomato brown rugose fruit virus emerges as the most dominant species, with a relative abundance exceeding 50% in both datasets. Intermediate versions, such as 2010 and 2015, also highlight Tobacco mosaic virus and Pepper mild mottle virus as prominent taxa in both methods.

While there are minor differences in the representation of less dominant species, such as Kluyveromyces lactis (strain WM37) and Pseudomonas aeruginosa, the overall patterns between MMseqs2 (Figure 24) and iMMseqs2 (Figure 25) remain remarkably similar. This consistency highlights the robustness of both methods in capturing key trends in species prevalence, while any slight variations can be attributed to the improved scoring and metadata integration in iMMseqs2. Together, these figures demonstrate that despite methodological refinements, both approaches provide comparable insights into shifts in species dominance over time, underscoring the reliability of the results.

The results of the Metacyc[2] label analysis reveal a relatively lower similarity between MMseqs2 (Figure 26) and iMMseqs2 (Figure 27) compared to the Swiss-Prot taxa results. This difference is likely attributable to the percentage data, which indicates that the top 10 results account for only 1% to 2% of the total outcomes. Such a low proportion may stem from the inherent characteristics of the dataset, specifically the complexity and diversity of labels associated with metabolic pathways. Additionally, iMMseqs2 focuses on detecting a wider variety of hits compared to the conventional method, potentially leading

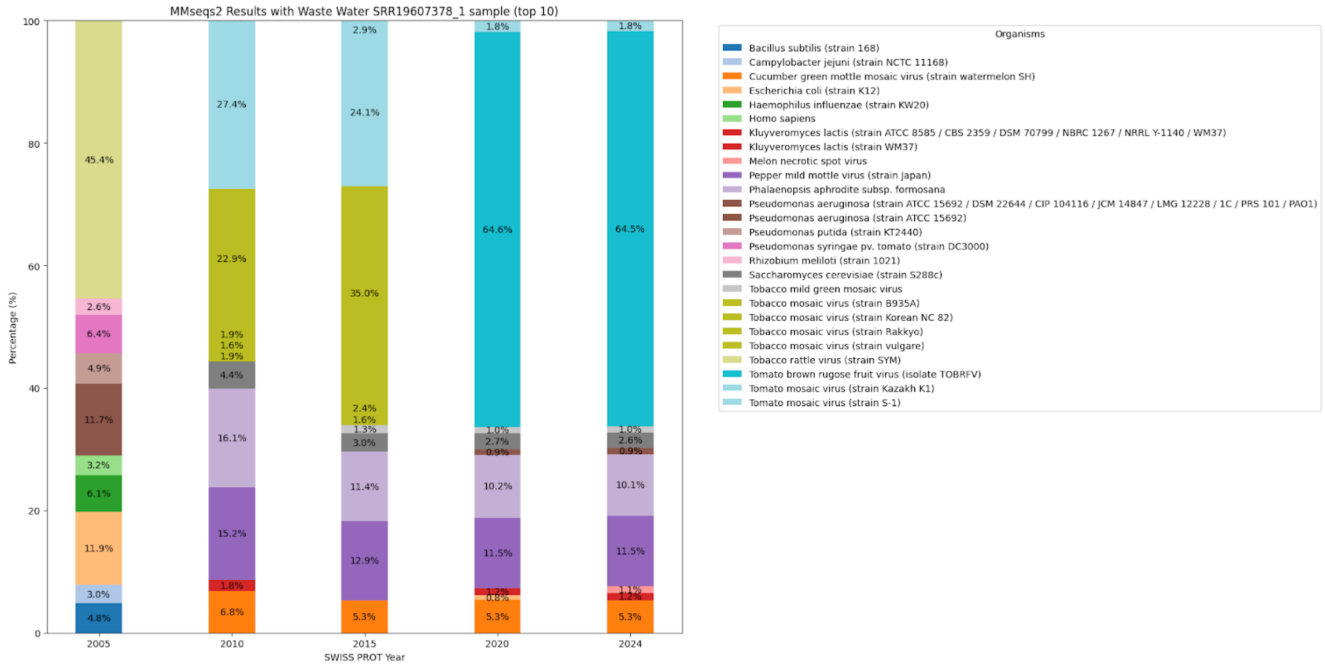

Figure 24. MMseqs2 Results with Waste Water SRR19607378_1 sample (top 10)

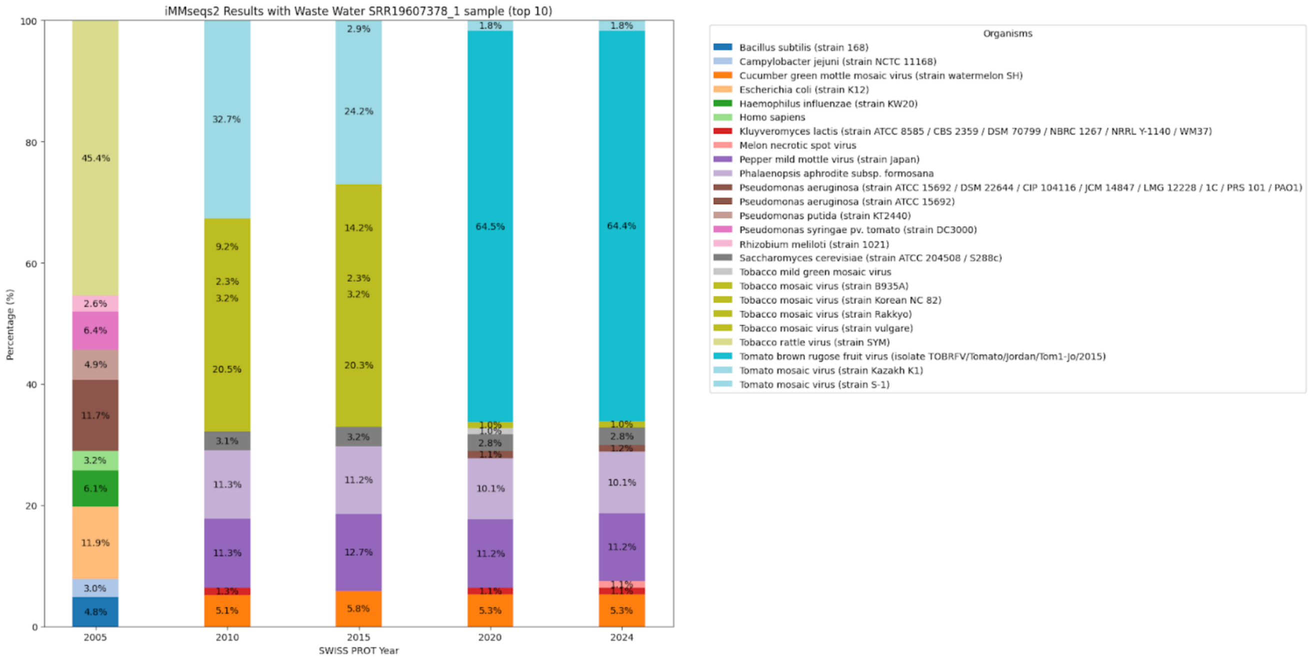

Figure 25. iMMseqs2 Results with Waste Water SRR19607378_1 sample (top 10)

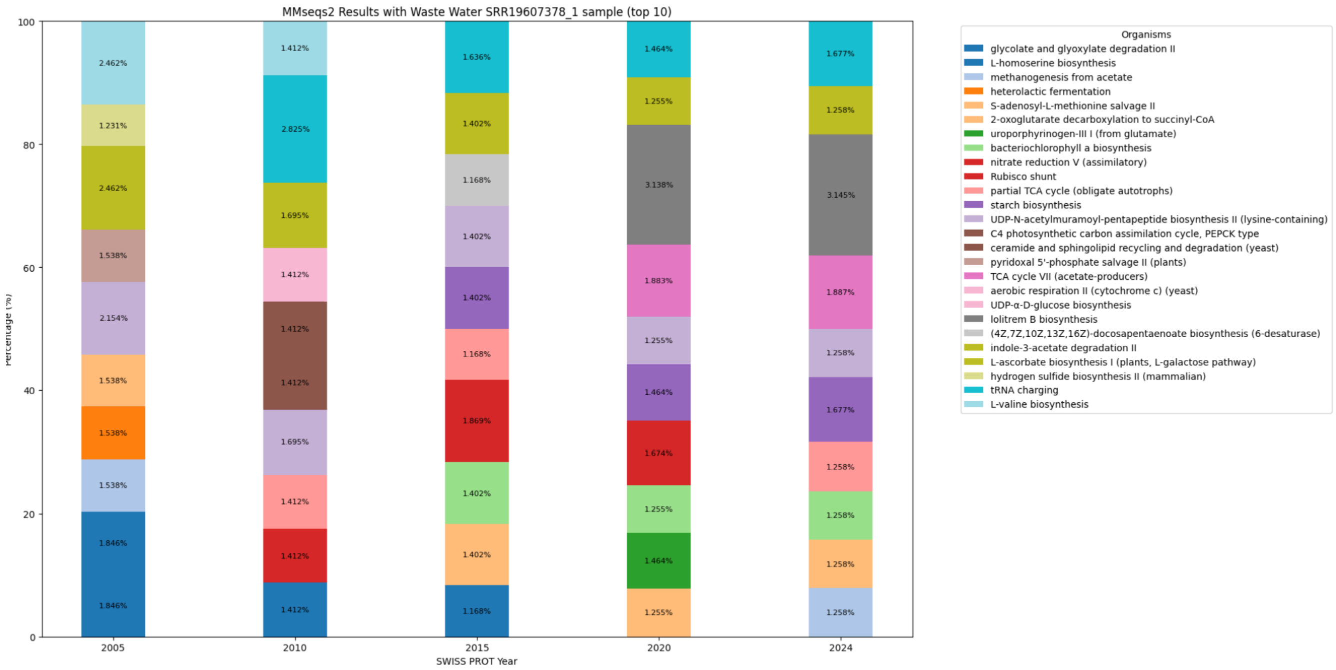

Figure 26. MMseqs2 metacyc Results with Waste Water SRR19607378_1 sample (top 10)

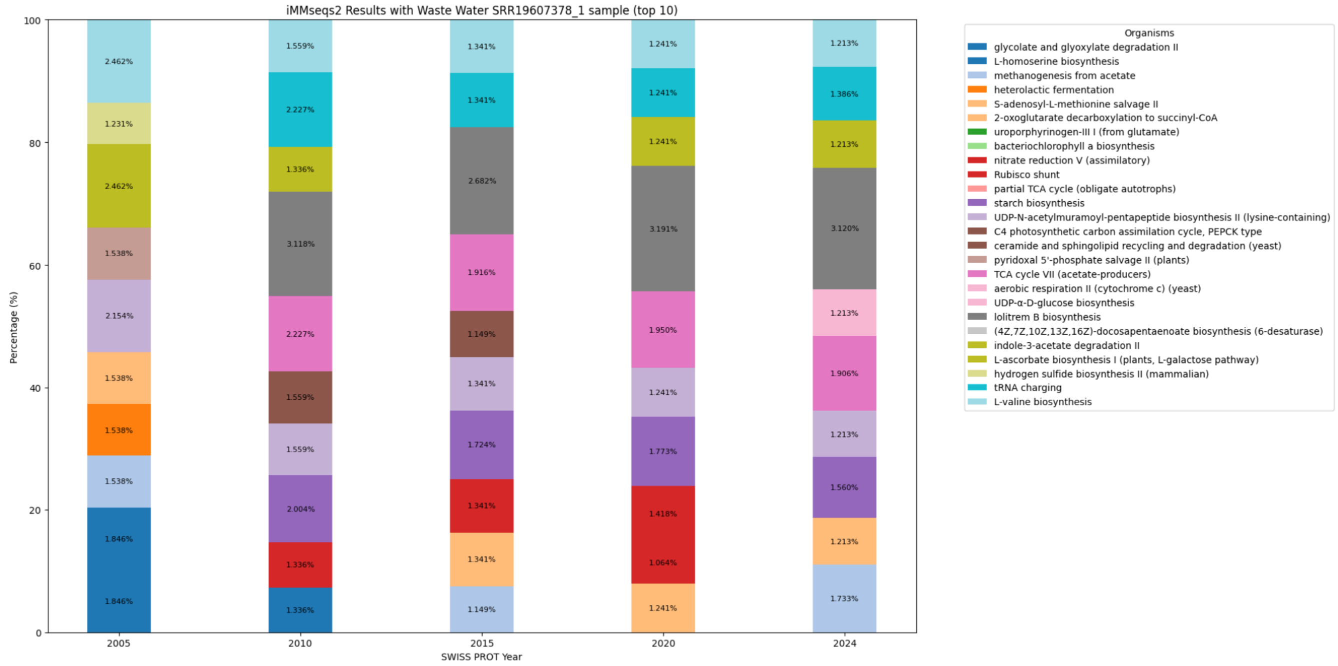

Figure 27. iMMseqs2 metacyc Results with Waste Water SRR19607378_1 sample (top 10)

to a lower concentration of top hits. These findings suggest that MMseqs2 and iMMseqs2 may provide complementary results in metabolic pathway analysis, thereby offering richer and more comprehensive biological insights.

10 m8e format

```
dblength:12417043
d2iyoa2 d2iz1a2 100.000 293 0   0   1   293 1   293 0.0 599
d2iyoa2 d2iz1b2 100.000 293 0   0   1   293 1   293 0.0 599
d7cb2b2 d7cb6b2 100.000 291 0   0   1   291 1   291 0.0 605
d7cb2b2 d7cb2c2 100.000 291 0   0   1   291 1   291 0.0 605
d2zygb2 d3fwna2 94.502  291 16  0   1   291 1   291 0.0 567
d2zygb2 d2zyaa2 94.483  290 16  0   1   290 1   290 0.0 566
d2jkvc2 d5uq9a2 100.000 293 0   0   1   293 1   293 0.0 613
d2jkvc2 d1pgpa1 94.613  297 16  0   1   297 1   297 0.0 591
d5uq9e2 d5uq9a2 100.000 292 0   0   1   292 1   292 0.0 610
```

Figure 28. Example of m8e file

Figure 28 shows an example of m8e format. The m8e format is identical to the m8 file format, except for the first line. The first line explicitly specifies the length of the database, eliminating the need to recalculate the database length during the merging function. This feature offers the advantage of improved processing speed.

References

1. Fox, N. K., Brenner, S. E. & Chandonia, J.-M. SCOPe: Structural classification of proteins—extended, integrating SCOP and ASTRAL data and classification of new structures. *Nucleic Acids Res.* **42**, D304–D309, 10.1093/nar/gkt1240 (2014).

2. Zhang, L. *et al.* Islet autoantibody seroconversion in type-1 diabetes is associated with metagenome-assembled genomes in infant gut microbiomes. *Nat. Commun.* **13**, 3551, 10.1038/s41467-022-31184-w (2022).
